# Predicting the Effects of CYP2C19 and Carboxylesterases on Vicagrel, a Novel P2Y12 Antagonist, by Physiologically Based Pharmacokinetic/Pharmacodynamic Modeling Approach

**DOI:** 10.3389/fphar.2020.591854

**Published:** 2020-12-08

**Authors:** Shuaibing Liu, Ziteng Wang, Xin Tian, Weimin Cai

**Affiliations:** ^1^ Department of Pharmacy, The First Affiliated Hospital of Zhengzhou University, Zhengzhou, China; ^2^ Department of Clinical Pharmacy, School of Pharmacy, Fudan University, Shanghai, China

**Keywords:** vicagrel, clopidogrel, CYP2C19, carboxylesterase, physiologically based pharmacokinetic/pharmacodynamic model

## Abstract

Vicagrel, a novel acetate derivative of clopidogrel, exhibits a favorable safety profile and excellent antiplatelet activity. Studies aim at identifying genetic and non-genetic factors affecting vicagrel metabolic enzymes Cytochrome P450 2C19 (CYP2C19), Carboxylesterase (CES) 1 and 2 (CES1 and CES2), which may potentially lead to altered pharmacokinetics and pharmacodynamics, are warranted. A physiologically based pharmacokinetic/pharmacodynamic (PBPK/PD) model incorporating vicagrel and its metabolites was constructed, verified and validated in our study, which could simultaneously characterize its sequential two step metabolism and clinical response. Simulations were then performed to evaluate the effects of *CYP2C19, CES1 and CES2* genetic polymorphisms as well as inhibitors of these enzymes on vicagrel pharmacokinetics and antiplatelet effects. Results suggested vicagrel was less influenced by CYP2C19 metabolic phenotypes and *CES1 428 G > A* variation, in comparison to clopidogrel. No pharmacokinetic difference in the active metabolite was also noted for volunteers carrying different *CES2* genotypes. Omeprazole, a CYP2C19 inhibitor, and simvastatin, a CES1 and CES2 inhibitor, showed weak impact on the pharmacokinetics and pharmacodynamics of vicagrel. This is the first study proposing a dynamic PBPK/PD model of vicagrel able to capture its pharmacokinetic and pharmacodynamic profiles simultaneously. Simulations indicated that genetic polymorphisms and drug-drug interactions showed no clinical relevance for vicagrel, suggesting its potential advantages over clopidogrel for treatment of cardiovascular diseases. Our model can be utilized to support further clinical trial design aiming at exploring the effects of genetic polymorphisms and drug-drug interactions on PK and PD of this novel antiplatelet agent.

## Introduction

Platelet P2Y_12_ receptor plays a crucial role in platelet activation. Physiologically, vessel damage stimulates the release of adenosine diphosphate (ADP) that binds to the P2Y_12_ receptor, which in turn leads to platelet activation and aggregation (Dorsam and Kunapuli, 2004; Cattaneo, 2015). P2Y_12_ receptor antagonists, e.g., thienopyridines bind to the P2Y_12_ receptor to block ADP-mediated platelet activation and aggregation.

Clopidogrel, a thienopyridine derivative, which targets P2Y_12_ receptor irreversibly, is widely used either alone or in combination with aspirin, remains a cornerstone of modern antiplatelet strategies (Hulot and Fuster, 2009). Clopidogrel is an inactive prodrug, requiring biotransformation to exhibit its antiplatelet effect. Only 15% of clopidogrel undergoes a two-step cytochrome P450 oxidation process including Cytochrome P450 2C19 (CYP2C19) to the pharmacologically active thiol metabolite H4 (AM-H4) via inactive intermediate metabolite 2-oxo-clopidogrel. While the majority is hydrolyzed by carboxylesterase 1 (CES1) to an inactive carboxylic acid derivative, which accounts for 85% of the clopidogrel-related compounds circulating in plasma (Sangkuhl et al., 2010). Furthermore, 2-oxo-clopidogrel and AM-H4 are also hydrolyzed by CES1 forming their respective inactive metabolites (Zhu et al., 2013).

Growing evidence suggests that about 5–40% of patients receiving conventional clopidogrel do not achieve adequate antiplatelet response (Matetzky et al., 2004). This well-known “clopidogrel resistance” phenomenon are attributed to *CYP2C19* null alleles *2 and/or *3, which are related with impaired enzymatic capacity of the two-step metabolism to AM-H4 and therefore declined clinical response (Kim et al., 2008).

Vicagrel, an acetate derivative of clopidogrel, was designed to overcome clopidogrel resistance (Shan et al., 2012). Like clopidogrel, it also undergoes a two-step metabolism process to form AM-H4 via 2-oxo-clopidogrel (Qiu et al., 2014). The difference is the enzymes that contribute to the first step of formation of 2-oxo-clopidogrel. Intestinal CES2 and arylacetamide deacetylase (AADAC) are the major enzymes responsible for the formation of 2-oxo-clopidogrel for vicagrel (Qiu et al., 2014; Jiang et al., 2017). Whereas, CYPs including CYP1A2, CYP2B6 and CYP2C19 expressed in the liver play dominant roles in metabolizing clopidogrel to 2-oxo-clopidogrel (Kazui et al., 2010). 2-oxo-clopidogrel is further metabolized by CYPs, i.e. CYP3A4, CYP2B6, CYP2C9 and CYP2C19 to form AM-H4, which is the same for both clopidogrel and vicagrel (Kazui et al., 2010; Zhang et al., 2020) (Supplementary Figure 1). Due to the much faster and more efficient formation of 2-oxo-clopidogrel in the gut than clopidogrel in the liver, it is anticipated to produce AM-H4 more efficiently and consistently than clopidogrel (Shan et al., 2012).

Vicagrel is now in Phase III development in China, suggesting the potential as a novel antiplatelet drug for cardiovascular diseases. Several clinical studies have demonstrated a favorable safety profile and excellent antiplatelet activity of vicagrel (Li et al., 2018; Liu et al., 2019; Zhang et al., 2020). Considering the involvement of carboxylesterases and CYP2C19 in vicagrel metabolism and the contribution of genetic variants and inhibitors of CYP2C19 and CES1 to the interindividual variability in clopidogrel pharmacokinetics and pharmacodynamics, further studies are still warranted to clarify such effects on vicagrel.

Physiologically based pharmacokinetic models (PBPK) have been widely used to predict the impact of pharmacogenetic factors and concomitant medication on drug exposure to support drug development and regulatory submissions. A further linkage to pharmacodynamic models (PBPK/PD) may allow better understanding and more realistic simulation regarding rational use in clinic (Rose et al., 2014). Therefore, the aim of the present study was to develop a mechanistic PBPK/PD model of the novel antiplatelet agent, vicagrel, to predict its inhibition on platelet aggregation (IPA) over time. The developed model was then used to evaluate the impacts of CYP2C19, CES1 and CES2 genetic variants and their respective inhibitors on the PK and PD of vicagrel, and to compare the predictions with clopidogrel.

## Methods

### Clinical Data Collection

A total of six available clinical studies of vicagrel and clopidogrel which determined AM-H4 plasma concentrations and/or IPA were included in this study [Trial 1 (Liu et al., 2015), Trial 2 (Angiolillo et al., 2011), Trial 3 (Simon et al., 2011), Trial 4 (Li et al., 2018), Trial 5 (Zhang et al., 2020) and Trial 6 (Tarkiainen et al., 2015)]. Detailed clinical trial information was described in Supplementary Table 1. Different dosing regimens, ethnicities and genetic polymorphisms were obtained from these studies. Plasma concentration-time and IPA-time profiles were digitized from figures in the publications using GetData Graph Digitizer 2.25 software (San Francisco, CA).

### Model Development

**FIGURE 1:**
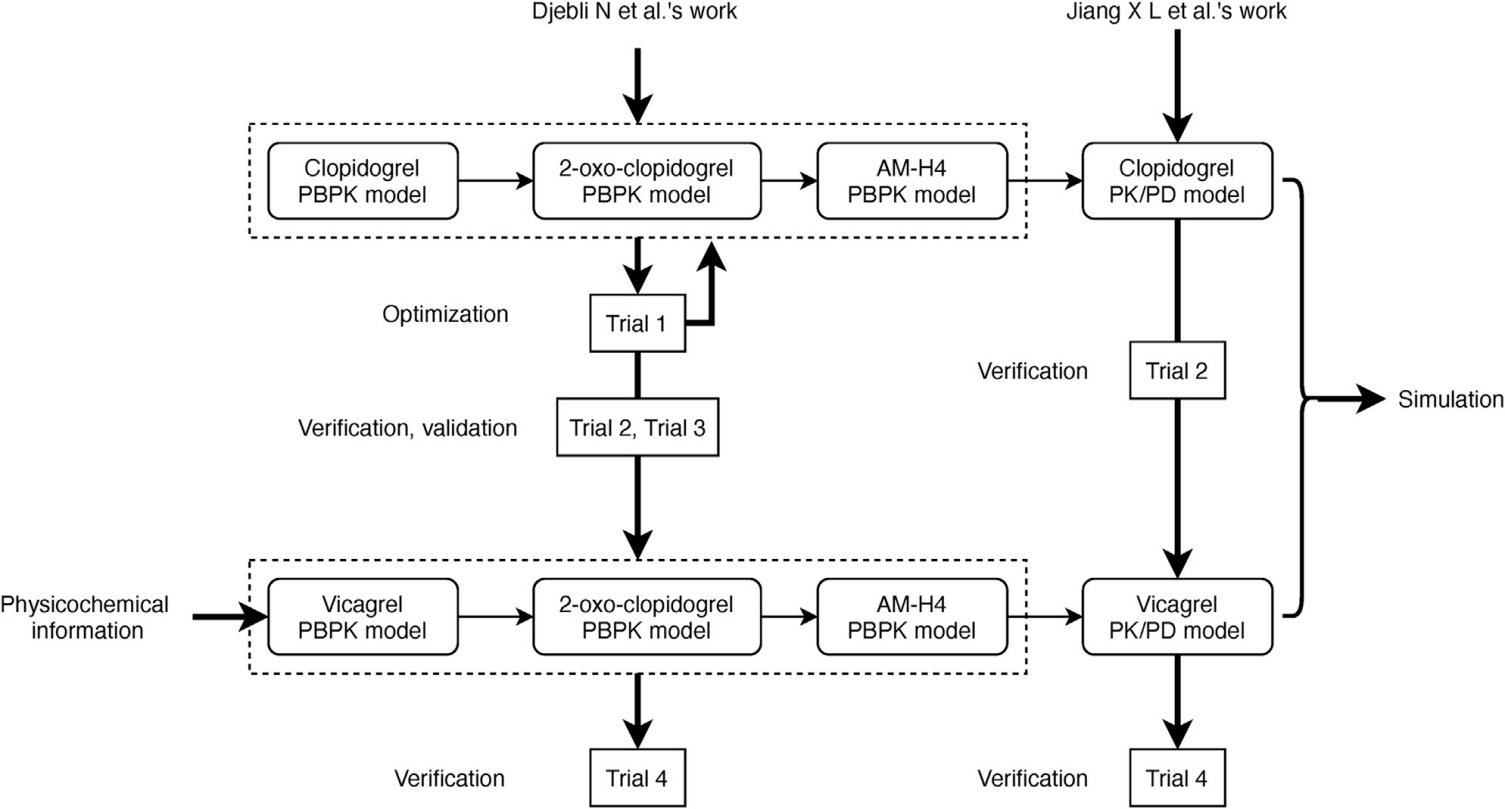
Scheme of development, verification, and validation of PBPK/PD models for vicagrel and clopidogrel.

PBPK/PD models of clopidogrel and vicagrel were developed utilizing Simcyp Simulator V19 software (a Certara company, United Kingdom, Sheffield). The overall scheme is presented in Figure 1. In brief, PBPK models for clopidogrel, 2-oxo-clopidogrel and AM-H4 were first built based on the work of Djebli et al. (Djebli et al., 2015). A mechanistic PD model of AM-H4 was then developed within Simcyp PD Custom module using Lua language and linked to PBPK model. PBPK model of vicagrel was constructed using a bottom-up approach. Key parameters for the PBPK models of the compounds are presented in Supplementary Table 2. The selection of parameters was based on available studies. Parameters for the PBPK model of clopidogrel and the two metabolites, 2-oxo-clopidogrel and AM-H4 were mainly obtained from Djebli et al.’s work (Djebli et al., 2015), and the enzymatic clearance of CES1 was incorporated based on Zhu et al.’s work. As a new drug, data for vicagrel were limited and those in our PBPK model were from manufacturer data and Jiang et al.’s work (Jiang et al., 2017). Parameters for the PD model of AM-H4 were from a population based study to describe the irreversible binding on P2Y12 receptors (Jiang et al., 2016). Models of omeprazole and simvastatin available in Simcyp compound library were directly used in the simulations.

PD model was based on the physiological process of active metabolite AM-H4, which irreversibly binds to the P2Y_12_ receptor on platelets and decreases platelet reactivity. The model was built to describe the time course between plasma concentration and 20 µM ADP-induced maximal platelet aggregation (MPA). A modified indirect response model was conducted to characterize the turnover of platelets and the irreversible inhibition.

$$\frac{dP}{dt}=k_{in}-P\times k_{out}-P\times C\times k_{irre}$$ (1)

where the rate of platelet formation (k_in_) and platelet degradation (k_out_) are assumed to be zero-order and first-order, respectively. k_irre_ is the second-order rate constant characterizing the AM-H4 mediated inactivation of platelets. P represents the turnover of platelets and C represents the molar concentration of AM-H4. Value for each parameter is presented in Supplementary Table 2.

Results were expressed as IPA, which was calculated by the following formula: IPA (%) =(MPA_0_−MPA_t_)/MPA_0_×100, where MPA_0_ is baseline MPA and MPA_t_ is MPA at time t.

### Simulations Using Pharmacokinetic/Pharmacodynamic Model

Simulations of clopidogrel and vicagrel were conducted in virtual healthy volunteers. A total of 100 individuals (10 × 10) were simulated during each trial. Healthy volunteers included in Simcyp were chosen for clinical studies including Caucasian volunteers and Chinese volunteers. For trials of CYP2C19 extensive metabolizers (EM), intermediate metabolizers (IM) or poor metabolizers (PM), frequency of the corresponding phenotype was modified to one in Simcyp Population tab. Dosage regimen used in the simulations were matched to each trial. Pharmacokinetic parameters were directly generated by Simcyp.

### Predicting the Effects of CYP2C19 and Carboxylesterases on Vicagrel Pharmacokinetics and Pharmacodynamics

The following scenarios were simulated to study the effects of genetic polymorphisms and inhibition regarding CYP2C19 or CESs enzyme on vicagrel. Pharmacokinetic and pharmacodynamic results were evaluated and compared with available data of vicagrel.

#### Effect of *CYP2C19* Genetic Polymorphism

Healthy Chinese volunteers with phenotypes of EM, IM or PM received a loading dose (LD) of 24 mg of vicagrel or 300 mg of clopidogrel on day 1 and daily maintenance dose (MD) of 6 mg of vicagrel or 75 mg of clopidogrel from day 2 to day 7.

#### Effect of CYP2C19 Inhibitor Omeprazole

CYP2C19 mechanism-based inhibitor omeprazole was used as the perpetrator drug. Simulation scenario was designed as Healthy Chinese volunteers administered 80 mg of omeprazole for 5 days and following with vicagrel of 24 mg LD and 6 mg/day MD for 4 days.

#### Effect of *Carboxylesterase 1* Genetic Polymorphism

A well-studied *CES1* single nucleotide polymorphism (SNP), g.*428 G > A* (rs71647871), which markedly decreased the catalytic efficiency of *CES1* *in vitro* (Hulot and Fuster, 2009), was investigated. The mutation lead to about 20% decrease in *CES1* activity based on a pop-PK analysis of clopidogrel (Angiolillo et al., 2011). Thus, enzymatic clearances of *CES1* 428 GA genotype for clopidogrel and 2-oxo-clopidogrel were then set to 80% of *CES1* 428 GG genotype, i.e., 240 μL/min/mg protein and 16 μL/min/mg protein, respectively, to reflect the impaired function. Vicagrel of 24 mg LD and 6 mg/day MD for 4 days was simulated in healthy Caucasian volunteers carrying different *CES1* g.*428 G > A* genotype considering the ethnic differences in mutant allele frequency, i.e., about 2–4% in white and 0% in Asian population (Simon et al., 2011).

#### Effect of *Carboxylesterase 1* Genetic Polymorphism

Two nonsynonymous SNPs of *CES2* reported in Japanese population, g.*100 C > T* (rs72547531) and g.*424 G > A* (rs72547532), which may cause functional alterations (Tarkiainen et al., 2015), were assessed in healthy Chinese volunteers. Enzymatic clearances of the CES2 functionally deficient alleles for vicagrel was reduced by 20-fold based on an *in vitro* study of irinotecan (Tarkiainen et al., 2015). Therefore, the impaired enzymatic clearance were set to 2,305 μL/min/mg protein. Dosage regimen of 24 mg LD and 6 mg/day MD for 4 days of vicagrel were simulated.

#### Effect of Carboxylesterases Inhibitor Simvastatin

CES1 and CES2 enzyme co-inhibitor simvastatin was utilized to study its inhibitory effect on pharmacokinetics and pharmacodynamics of vicagrel. Reversible inhibition constants (K_i_) of simvastatin on CES1 and CES2 were 0.11 and 0.67 µM, respectively (Fukami et al., 2010). Dosage regimen are designed as follows: simvastatin 80 mg/day was given for five consecutive days, on day 6 vicagrel were co-administered with simvastatin. Sensitivity analysis function within Simcyp was then performed to investigate the contributions of inhibitory potential of CES1 and CES2 to vicagrel pharmacokinetic profiles.

## Results

### Physiologically Based Pharmacokinetic Model Development, Verification, and Validation

PBPK models for clopidogrel and its two metabolites were constructed based on the work of Djebli N et al. (Djebli et al., 2015) and optimized using our previous data obtained from healthy Chinese volunteers (Trial 1). Minimal PBPK model was applied for clopidogrel to reduce model complexity (Tornio et al., 2014) and fit the data better. Additional clearances of clopidogrel and 2-oxo-clopidogrel attributed to CES1 mediated enzymatic clearances were set based on Zhu et al.’s work (Zhu et al., 2013). User-defined esterase enzyme in Simcyp was selected to represent AADAC-mediated metabolism. Relative enzyme abundance and kinetic parameters information of CES2 and AADAC were obtained from Jiang et al. and Vrana, M. ’s work (Jiang et al., 2017; Vrana and Prasad, 2019).

**FIGURE 2:**
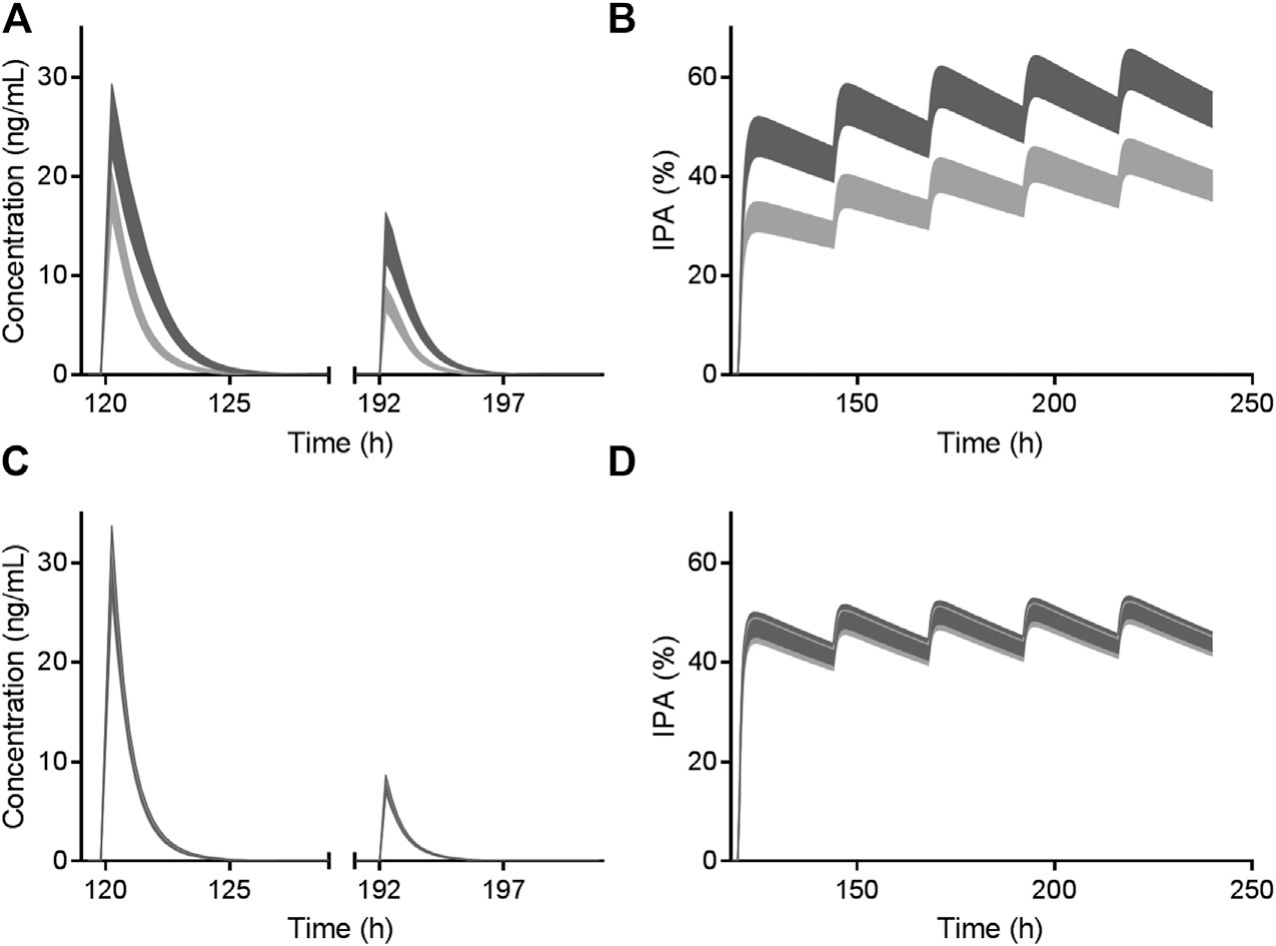
Simulated AM-H4 concentration **(left row)** and IPA **(right row)** vs. time of clopidogrel **(A and B)** and vicagrel **(C and D)** co-administrated with omeprazole. Dosage regimens were 300 mg LD and 75 mg/day MD for 4 days of clopidogrel and 24 mg LD and 6 mg/day MD for 4 days of vicagrel, respectively. Bands represent simulated 95% confidence interval in the presence (light gray)/absence (dark gray) of 80 mg omeprazole treatment. *X* axis was set according to the first dose of omeprazole.

**FIGURE 3:**
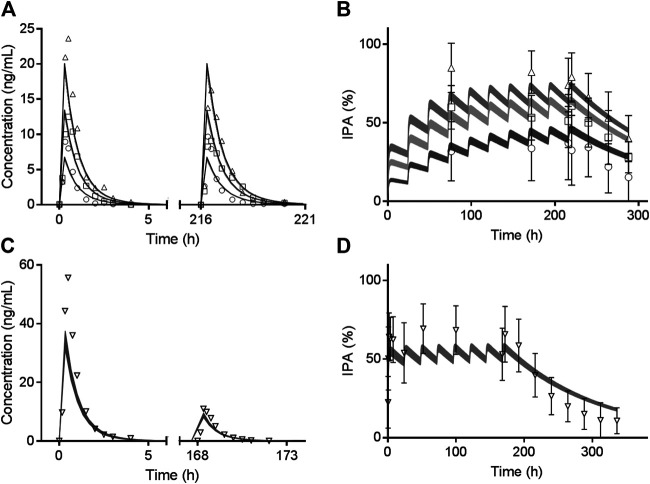
Observed mean value (symbols) and simulated 95% confidence interval (bands) of AM-H4 concentration **(left row)** and IPA **(right row)** vs. time following different doses of vicagrel according to Trial 4. Different shades of gray bands refer to corresponding observed values of different doses. Observed IPAs are presented as mean ± SD.

Trial 2 was used for clopidogrel model verification and validation. Trial 3 and Trial 5 including CYP2C19 phenotyped healthy volunteers were used to verify the effects of *CYP2C19* genetic polymorphisms on AM-H4. Models of two metabolites were then utilized for vicagrel, which was also verified by Trial 4 and Trial 5. All the 74 available fold-errors of C_max_ and AUC_0-t_ met a criteria of less than 2, while 67 of which were less than 1.5 (Supplementary Table 3). Simulated concentrations of AM-H4 metabolized from clopidogrel based on Trial 2 are illustrated in Figure 2A. Simulated and observed concentrations of AM-H4 metabolized from vicagrel based on Trial 4 are presented in left row Figure 3. Results suggested good predictivity of AM-H4 under different dose regimens, genetic polymorphisms and ethnics. Models of omeprazole and simvastatin were also verified by published data (Backman et al., 2000; Baldwin et al., 2008). Simulated and observed pharmacokinetic parameters are provided in Supplementary Table 4.

### Pharmacokinetic/Pharmacodynamic Model Development and Verification

Simulated IPA profiles of clopidogrel based on Trial 2 are shown in Figure 2B. Simulated and observed IPA profiles of vicagrel based on Trial 4 are presented in right row of Figure 3. Recovery time of platelet function after vicagrel discontinuation was around 7 days, suggesting a comparable irreversible inhibition behavior to clopidogrel. Entire time course of IPA was fully captured by our PD model, which was consistent with the pharmacological mechanism of the thienopyridine antiplatelet agents (Sangkuhl et al., 2011).

### Effects of Genetic Polymorphisms and Inhibitor of CYP2C19 on Vicagrel Pharmacokinetics and Pharmacodynamics

**FIGURE 4:**
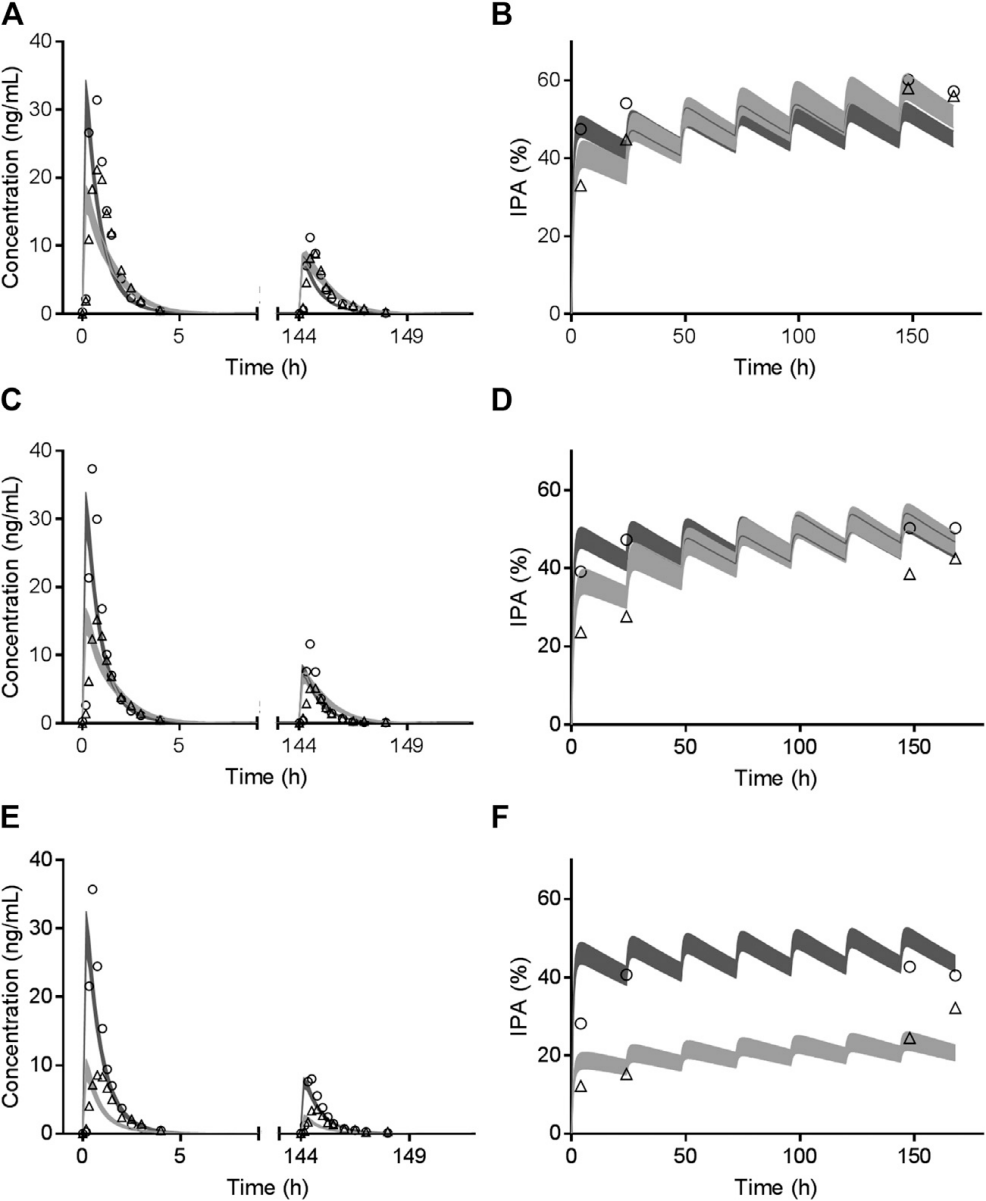
Observed mean value (symbols) and simulated 95% confidence interval (bands) of AM-H4 concentration **(left row)** and IPA **(right row)** vs. time among CYP2C19 EM **(A and B)**, IM **(C and D)** and PM **(E and F)** population receiving clopidogrel or vicagrel.

Results in term of CYP2C19 phenotypes on vicagrel and clopidogrel according to Trial 5 are shown in Supplementary Table 3 and Figure 4. Comparable *in vivo* exposure of AM-H4 and IPA between vicagrel and clopidogrel were observed in EM subjects, especially during MD phase. Remarkable decrease was noted for clopidogrel in PM subjects, while those for vicagrel were less influenced by *CYP2C19* polymorphisms.

As illustrated in Figure 2, vicagrel was also less affected by CYP2C19 inhibitor omeprazole compared to clopidogrel. A long-term treatment of omeprazole resulted in only slightly decrease in AM-H4 concentration and IPA, when a dosage regimen of 24 mg LD and 6 mg MD was given.

### Effects of Genetic Polymorphisms and Inhibitor of Carboxylesterases on Vicagrel Pharmacokinetics and Pharmacodynamics

**FIGURE 5:**
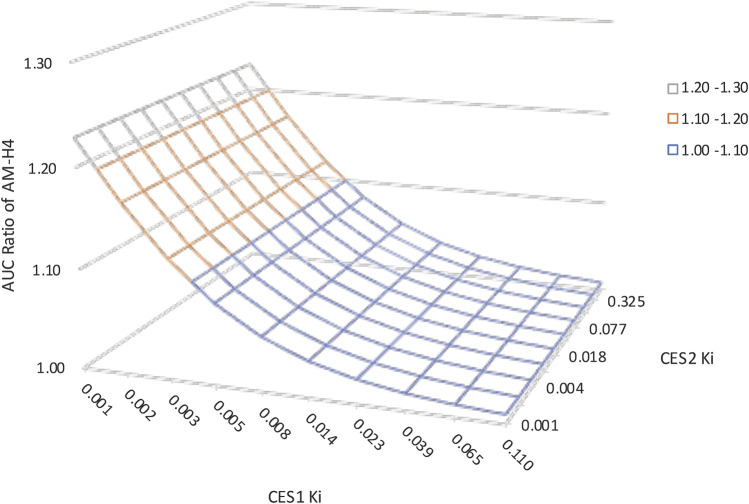
Sensitivity analysis suggesting the impact of carboxylesterases K_i_ of simvastatin on vicagrel predicted AUC_0-t_ ratios. K_i_ on CES1 ranged from 0.001–0.11. K_i_ on CES2 ranged from 0.001–0.67.

Table 1 summarized the effects of genetic polymorphisms and inhibitors of CESs on pharmacokinetic parameters of vicagrel and AM-H4. A slightly increase in AM-H4 exposure and subsequently elevated IPA response were observed for *CES1 428 G/A* carriers (Table 1, Supplementary Figure 2). Although proportion of change up to more than 300-fold was observed for parameters of vicagrel among volunteers carrying *CES2* defect alleles, ratios of pharmacokinetic parameters for AM-H4 were approximately 1. Similarly, no difference of AM-H4 parameters was observed when volunteers were co-administrated with simvastatin. Overall, genetic polymorphisms and inhibitor of CESs may not result in clinically relevant difference of pharmacodynamic profiles of vicagrel. Further analysis of K_i_ of simvastatin on CES1 and CES2 in terms of AM-H4 pharmacokinetics was performed using sensitivity analysis in a range of 0.001–0.11 and 0.001–0.67, respectively. As illustrated in Figure 5, AUC_0-t_ ratio of AM-H4 increased as the K_i_ values decreased, and sensitivity to K_i_ on CES1 is greater when the value is low.

**TABLE 1:** Comparison of pharmacokinetic parameters of vicagrel and AM-H4 in the presence/absence of various factors regarding genetic polymorphisms and inhibitor of CESs.

Populations	Simulated analytes	Route	Groups	Parameters for the first dose				Parameters for the last dose			
				AUC_0-24_ (ng·h/mL)	Ratio of mean AUC_0-24_	C_max_ (ng/ml)	Ratio of mean C_max_	AUC_0-24_ (ng·h/mL)	Ratio of mean AUC_0-24_	C_max_ (ng/ml)	Ratio of mean C_max_
Caucasian	Vicagrel	Oral	*CES1* G/G	0.09 ± 0.07	1.00	0.09 ± 0.07	1.00	0.02 ± 0.02	1.00	0.02 ± 0.02	1.00
			*CES1* G/A	0.09 ± 0.07		0.09 ± 0.07		0.02 ± 0.02		0.02 ± 0.02	
	AM-H4		*CES1* G/G	36.10 ± 14.00	0.89	36.10 ± 15.94	0.89	9.10 ± 3.52	0.89	9.17 ± 4.05	0.90
			*CES1* G/A	40.48 ± 14.96		40.42 ± 17.12		10.20 ± 3.76		10.24 ± 4.34	
Chinese	Vicagrel	Oral	*CES2* wild type	0.12 ± 0.08	0.003	0.10 ± 0.07	0.003	0.03 ± 0.02	0.003	0.03 ± 0.02	0.004
			*CES2* defect allele	40.46 ± 27.27		29.29 ± 16.61		10.11 ± 6.82		7.32 ± 4.15	
	AM-H4		*CES2* wild type	31.35 ± 13.07	1.00	31.54 ± 15.19	1.03	7.94 ± 3.31	1.00	8.05 ± 3.86	1.03
			*CES2* defect allele	31.41 ± 13.09		30.57 ± 14.84		7.95 ± 3.31		7.80 ± 3.77	
Chinese	Vicagrel	Oral	w/o simvastatin	0.12 ± 0.08	0.80	0.10 ± 0.07	0.71	0.03 ± 0.02	0.75	0.03 ± 0.02	0.75
			w/simvastatin	0.15 ± 0.10		0.14 ± 0.09		0.04 ± 0.03		0.04 ± 0.02	
	AM-H4		w/o simvastatin	28.57 ± 11.55	0.99	29.17 ± 14.95	1.00	7.23 ± 2.93	1.00	7.45 ± 3.80	1.00
			w/simvastatin	28.84 ± 11.63		29.22 ± 14.94		7.30 ± 2.95		7.46 ± 3.80	

Parameters are presented as mean ± SD.

Each ratio of mean value was calculated from the upper mean value divided by lower mean value of each parameter.

## Discussion

Our work is the first accurately and simultaneously describing the pharmacokinetics of two parent drugs of antiplatelet agents, their primary and secondary metabolites and pharmacodynamics via a full dynamic PBPK/PD modeling approach. A combined PBPK/PD model was firstly built for clopidogrel based on a published PBPK model describing clopidogrel sequential metabolism (Djebli et al., 2015) and a pop-PD model (Jiang et al., 2016). Vicagrel PBPK/PD model was then constructed by linking the parent drug to the metabolites.

The PK profiles of vicagrel, clopidogrel, their common metabolites 2-oxo-clopidogrel and AM-H4 were characterized via a bottom-up approach integrating available physicochemical and *in vitro* absorption, distribution, metabolism and excretion information. The models were successfully verified and validated for clopidogrel and AM-H4 but not vicagrel and 2-oxo-clopidogrel because of the difficulty of determination in plasma and limited clinical data (Hua et al., 2015; Liu et al., 2019). The irreversible inhibition of platelet aggregation of thienopyridines was characterized by an indirect response model and linked to plasma concentrations of AM-H4. Interindividual variability was considered in k_in_, k_out_, k_irr_ and MPA_0_ based on estimated results (Jiang et al., 2016). Similar to clopidogrel, slow loss of inhibition could be observed after vicagrel discontinuation because of the irreversible binding of AM-H4 to P2Y_12_ receptor.

For clopidogrel, simulation results of the effects of omeprazole and CYP2C19 phenotypes on its pharmacokinetics and pharmacodynamics based on Trial 2, Trial 3 and Trial 5 verified the important role of CYP2C19 play in clopidogrel metabolic processes. Regarding vicagrel, less impacts of both CYP2C19 inhibitor and phenotypes were observed on pharmacokinetics and subsequent pharmacodynamics. Since CYP2C19 still participated in the second step of vicagrel metabolism (Qiu et al., 2014; Jiang et al., 2017), AM-H4 exposure and IPA slightly declined in PM volunteers comparing with those in EM and IM volunteers (Supplementary Table 3 and Figure 4), which might not be clinically relevant. The results confirmed the assumption in previous studies that dosage adjustment or alternative therapy was unnecessary for patients identified as CYP2C19 PM phenotype receiving vicagrel treatment (Zhang et al., 2020).

Most of clopidogrel was hydrolyzed by CES1 to the inactive carboxylic acid metabolite. CES1 was also involved in the metabolism of 2-oxo-clopidogrel and AM-H4. An *in vitro* study reported that enzymatic activity of the CES1 variant c.428 G > A was completely abolished in terms of catalyzing the hydrolysis of clopidogrel and 2-oxo-clopidogrel (Zhu et al., 2013). An *in vivo* study confirmed increased AM-H4 concentration and antiplatelet effect in *428 G/A* heterozygotes, resulting from impaired hydrolysis of clopidogrel (Trial 6) (Tarkiainen et al., 2015). A pop-PK analysis suggested around 80% of remained CES1 activity for heterozygotes (Jiang et al., 2016). It was then incorporated into our model. Our simulated results showed less impact of *CES1* genetic polymorphisms on vicagrel with respect to both PK and PD, when compared to clopidogrel of Trial 6 (Supplementary Figure 1).

*CES1A2 -816A > C* is another genetic polymorphism of CES1, which is common in Chinese population with allele frequency of around 25%. But conflicting results remain as to the effect on clopidogrel antiplatelet response. Zou et al. reported this mutant was associated with greater platelet response to clopidogrel (Zou et al., 2014), while Xie et al. observed attenuated platelet reactivity to clopidogrel (Xie et al., 2014). Therefore, the impact of this SNP on vicagrel pharmacokinetics and pharmacodynamics was not explored in the current study.

Another major isoform of human carboxylesterase, CES2, was reported to catalyze vicagrel to form its intermediate metabolite, 2-oxo-clopidogrel (Qiu et al., 2016). Subsequent study found AADAC in the human intestine was also involved in the hydrolytic metabolism of vicagrel with a contribution of approximately 53% (Jiang et al., 2017). AADAC, also known as CES5A1, is responsible for the hydrolysis of flutamide, phenacetin and rifampicin (Shimizu et al., 2014). Comparable intestinal protein expressions and enzyme affinities for vicagrel between AADAC and CES2 were determined (Jiang et al., 2017; Vrana and Prasad, 2019). Since AADAC was not considered in the current version of Simcyp software, a user-defined esterase enzyme was selected to represent the contribution of AADAC to the hydrolysis of vicagrel in our PBPK model. The AADAC*2 allele (g.13651 G > A, rs1803155) and AADAC*3 allele (g.13651 > A/g.14008T>C) are two most common SNPs which are reported to be associated with reduced enzyme activity *in vitro* and *in vivo* (Shimizu et al., 2012; Sloan et al., 2017; Francis et al., 2019). It would be interesting to explore the effect of AADAC genetic polymorphisms on vicagrel activation and subsequent antiplatelet response. However, due to the lack of absolute protein quantitation data for different genotypes, enzymatic alteration resulting from genetic polymorphisms was not further explored in our study. Further studies toward determining the absolute protein levels should be underway.

Large ethnic differences in *CES2* alleles frequencies have been documented across populations (Marsh et al., 2004). The two nonsynonymous SNPs, *CES2* g. 100C > T and *CES2* g.424 G > A found in Japanese population were investigated in our study (Kim et al., 2003). Although elevated protein expression levels were observed for both variants *in vitro*, further studies indicated almost complete loss of carboxylesterase activity toward irinotecan and declined *in vivo* exposure of its metabolites SN-38 and SN-38G (Kubo et al., 2005). Our simulation also found increased vicagrel concentrations due to the decreased catalytic efficiency of CES2, but the variations resulted in almost no change of AM-H4 concentration. It is not surprising since CES2 contributed to only one step of the sequential metabolism of vicagrel, the decreased enzyme activity caused by CES2 variants may be partly compensated by AADAC.

Simvastatin, an oral cholesterol-lowering medication, showed strong inhibitory effects on imidapril hydrolase activity by CES1 and irinotecan hydrolase activity by CES2 *in vitro* (Fukami et al., 2010). Another study demonstrated that simvastatin could significantly inhibit CES1-mediated hydrolysis of clopidogrel, 2-oxo-clopidogrel and AM-H4, while the production of AM-H4 was not affected (Wang et al., 2015). Unlike clopidogrel, an *in vitro* study has identified declined AM-H4 production from vicagrel when co-incubated with simvastatin (Jiang et al., 2017), but no difference of AM-H4 concentrations was observed in our simulations. It could be partly explained as the rapid elimination property of both perpetrator drug and victim drug that the effective inhibitory potential could not be reached.

Study of sensitivity analysis to K_i_ values suggested a more pivotal role of CES1 in the alteration of esterase enzymatic function regarding AM-H4 pharmacokinetics, because CES1 was directly related to the formation of AM-H4. The result was consistent with the above study of the effect of carboxylesterases genetic polymorphisms that *CES1* defect allele could result in more obviously decreased AM-H4 exposure. Since parameter sensitivity to K_i_ on CES1 is greater when the value is low, drug-drug interaction studies could be utilized between vicagrel and several clinical medications which were reported to be more potent CES1 inhibitors, e.g., telmisartan, nitrendipine (Wang et al., 2018), to support drug development and treatment optimization for this novel antiplatelet agent.

Due to the low plasma concentrations of vicagrel parent drug (Liu et al., 2019), the PBPK model was unable to be verified, which may result in bias when evaluating the intestinal first-pass metabolism of vicagrel. Additionally, although all the simulated pharmacokinetic parameters for vicagrel-mediated AM-H4 fell within the acceptance criteria based on two published studies of long-term treatment (Trial 4 and Trial 5), our results were much lower than those of a single-ascending-dose study of vicagrel (Liu et al., 2019) (Supplementary Table 5). A better optimization of vicagrel model and more clinical data are necessary to help characterize the biotransformation of vicagrel.

In conclusion, a PBPK/PD model for a novel antiplatelet agent, vicagrel, was presented in our study, which could capture the PK and PD profiles simultaneously. The impacts of genetic polymorphisms and inhibitors of CYP2C19 and carboxylesterases were then evaluated from both pharmacokinetic and pharmacodynamic viewpoints. Vicagrel was less influenced by these factors when compared to clopidogrel, suggesting the potential as a novel antiplatelet agent. Our model can be successfully used to facilitate optimal treatment plan of vicagrel for cardiovascular diseases.

## Data Availability

The original contributions presented in the study are included in the article/Supplementary Material, further inquiries can be directed to the corresponding authors.
