# Corrigendum: Predicting the Effects of CYP2C19 and Carboxylesterases on Vicagrel, a Novel P2Y12 Antagonist, by Physiologically Based Pharmacokinetic/Pharmacodynamic Modeling Approach

**DOI:** 10.3389/fphar.2021.668861

**Published:** 2021-04-16

**Authors:** Shuaibing Liu, Ziteng Wang, Xin Tian, Weimin Cai

**Affiliations:** ^1^ Department of Pharmacy, The First Affiliated Hospital of Zhengzhou University, Zhengzhou, China; ^2^ Department of ClinicalPharmacy, School of Pharmacy, Fudan University, Shanghai, China

**Keywords:** vicagrel, clopidogrel, CYP2C19, carboxylesterase, physiologically based pharmacokinetic/pharmacodynamic model

In the published article, there was an error in the Funding statement. The correct Funding appears below.

“This work was supported by the National Natural Science Foundation of China (Grant No. 81603204) and National Science and Technology Major Project of China (Grant No.2020ZX09201-009)”.

The authors apologize for this error and state that this does not change the scientific conclusions of the article in any way. The original article has been updated.

