# Supplementary material for: Predicting the Effects of CYP2C19 and Carboxylesterases on Vicagrel, a Novel P2Y12 Antagonist, by Physiologically Based Pharmacokinetic/Pharmacodynamic Modeling Approach
Source: Front Pharmacol. 2020 Dec 8;11:591854. doi: 10.3389/fphar.2020.591854 (PMC7793822; doi:10.3389/fphar.2020.591854)
Supplement: Supplementary file 1 [file datasheet1.docx]

Supplementary Table 1 Detailed information of clinical trials used for modeling and simulation

| Trials | Populations | Subject number | Genotypes/Phenotypes | Drugs | Doses and regimens | Route | References |
| --- | --- | --- | --- | --- | --- | --- | --- |
| 1 | Chinese | 11 | NA | clopidogrel | 300 mg single dose | oral | (1) |
| 2 | Caucasian | 65 | NA | clopidogrel | 300 mg D1 + 75 mg D2 to D5 | oral | (2) |
| 3 | Caucasian | 10 | CYP2C19 EM, IM or PM | clopidogrel | 300 mg D1 + 75 mg D2 to D5 | oral | (3) |
| 4 | Chinese | 9 | NA | clopidogrel | 75 mg QD 10 days | oral | (4) |
|  |  | 9 | NA | vicagrel | 5 mg QD 10 days |  |  |
|  |  | 9 | NA | vicagrel | 10 mg QD 10 days |  |  |
|  |  | 9 | NA | vicagrel | 15 mg QD 10 days |  |  |
|  |  | 15 | NA | vicagrel | 30 mg D1 + 7.5 mg D2 to D8 |  |  |
| 5 | Chinese | 16 | CYP2C19 EM, IM or PM | clopidogrel | 300 mg D1 + 75 mg D2 to D7 | oral | (5) |
|  |  | 16 | CYP2C19 EM, IM or PM | vicagrel | 24 mg D1 + 6 mg D2 to D7 |  |  |
| 6 | Finnish | 12 | *CES1 G/G* or *G/A* | clopidogrel | 600 mg single dose | oral | (6) |

Supplementary Table 2 Physicochemical, *in vitro* pharmacokinetic and estimated pharmacodynamic information used for PBPK/PD modeling of clopidogrel and vicagrel

|  | Parameters | Units | Values implemented in Simcyp | | | | | | | |
| --- | --- | --- | --- | --- | --- | --- | --- | --- | --- | --- |
| *PK model* |  |  |  |  |  |  |  |  |  |  |
|  |  |  | Clopidogrel | | Vicagrel | | 2-oxo-clopidogrel | | AM-H4 | |
| Modules |  |  | Value | Reference | Value | Reference | Value | Reference | Value | Reference |
| PhysChem and Blood Binding | MW | g/mol | 321.8 |  | 379.9 | Manufacturer data | 337.8 |  | 355.8 |  |
|  | logP_o:w_ |  | 3.89 |  | 1.89 | Manufacturer data | 2.96 |  | 3.6 |  |
|  | pKa |  | 4.55 |  | 4.525 | Manufacturer data | 3.41 |  | 3.20, 5.10 |  |
|  | fu |  | 0.02 |  | 0.07 | Predicted by QSAR | 0.03 |  | 0.018 |  |
| Absorption | Model |  | First order |  | First order |  |  |  |  |  |
|  | P_eff, man_ | 10^-4^ cm/s | 0.466 |  | 3.38 | Predicted by PSA |  |  |  |  |
| Distribution | Model |  | Minimal PBPK |  | Minimal PBPK |  | Minimal PBPK |  | Minimal PBPK |  |
|  | Vss | L/kg | 5 | Tornio A et al.(7) | 0.13 | Predicted by Method 2 | 0.1 |  | 0.23 |  |
| Elimination | Enzyme System |  | Recombinant |  |  |  | Recombinant |  |  |  |
|  | CYP1A2 V_max_ | pmol/min/pmol isoform | 2.27 |  |  |  |  |  |  |  |
|  | CYP1A2 K_m_ | μM | 1.58 |  |  |  |  |  |  |  |
|  | CYP2B6 V_max_ | pmol/min/pmol isoform | 7.66 |  |  |  | 2.48 |  |  |  |
|  | CYP2B6 K_m_ | μM | 2.08 |  |  |  | 1.62 |  |  |  |
|  | CYP2C9 V_max_ | pmol/min/pmol isoform |  |  |  |  | 0.855 |  |  |  |
|  | CYP2C9 K_m_ | μM |  |  |  |  | 18.1 |  |  |  |
|  | CYP2C19 V_max_ | pmol/min/pmol isoform | 7.52 |  |  |  | 9.06 |  |  |  |
|  | CYP2C19 K_m_ | μM | 1.12 |  |  |  | 12.1 |  |  |  |
|  | CYP3A4 V_max_ | pmol/min/pmol isoform |  |  |  |  | 3.63 |  |  |  |
|  | CYP3A4 K_m_ | μM |  |  |  |  | 27.8 |  |  |  |
|  | Enzyme System |  | Microsomal |  | Recombinant |  | Microsomal |  |  |  |
|  | CES1 Cl_int_ | μL/min/mg protein | 300 | Modified based on Zhu HJ et al.(8) |  |  | 20 | Modified based on Zhu HJ et al.(8) |  |  |
|  | CES2 Cl_int_ | μL/min/mg protein |  |  | 46100 | Jiang J et al.(9) |  |  |  |  |
|  | User ES Cl_int_ | μL/min/mg protein |  |  | 39000 |  |  |  |  |  |
|  | *In vivo* clearance  CL_po_ | L/h |  |  |  |  |  |  | 400 | Modified |
| *PD model* |  |  |  |  |  |  |  |  |  |  |
|  |  |  | Value | SE (%) | Reference |  |  |  |  |  |
|  | k_in_ | 1/h | 0.00724 | 7.8 | Jiang XL et al.(10) |  |  |  |  |  |
|  | k_out_ | 1/h | 0.00724 | 7.8 |  |  |  |  |  |  |
|  | k_irre_ | 1/μM/h | 62.4 | 4.5 |  |  |  |  |  |  |
|  | MPA_0_ | % | 75.8 | 2 |  |  |  |  |  |  |

Parameters were obtained from Djebli N et al’s model (11) unless otherwise stated in the table.

Cl_int_: intrinsic clearance; CL_po_: oral clearance; k_in_: rate of platelet formation; k_irre_: rate of AM-H4 mediated inactivation of platelets; k_out_: rate of platelet degradation; MPA_0_: 20 µM ADP-induced maximal platelet aggregation at baseline; P_eff, man_: effective human jejunum permeability; PSA: polar surface area; QSAR: Quantitative Structure–Activity Relationship.

Supplementary Table 3 Clinical study data used in verification and validation of PBPK models of clopidogrel and vicagrel and comparison to simulated results

| Trials | Genotypes | Drugs | Determined analytes | AUC_0-t_ (ng·h/mL) | | | C_max_ (ng/mL) | | |
| --- | --- | --- | --- | --- | --- | --- | --- | --- | --- |
|  |  |  |  | Observed | Simulated | Ratio of mean AUC_0-t_  (Observed/ Simulated) | Observed | Simulated | Ratio of mean  C_max_ (Observed/ Simulated) |
| *Parameters for the first dose* | | |  |  |  |  |  |  |  |
| 1 | NA | clopidogrel | clopidogrel | 10.6±3.73 | 12.0±11.6 | 0.89 | 3.84±1.26 | 5.17±6.22 | 0.74 |
| 2 | NA | clopidogrel | clopidogrel | 10.4±10.5 | 10.9±7.70 | 0.96 | 5.24±6.29 | 5.90±7.26 | 0.89 |
| 2 | NA | clopidogrel | AM-H4 | 49.4±36.7 | 42.5±32.8 | 1.16 | 38.5±21.6 | 26.2±17.6 | 1.47 |
| 2 | NA | clopidogrel+omeprazole | clopidogrel | 13.6±15.5 | 12.1±9.50 | 1.12 | 5.56±7.92 | 6.41±8.44 | 0.87 |
| 2 | NA | clopidogrel+omeprazole | AM-H4 | 26.2±14.1 | 22.7±15.7 | 1.15 | 20.6±9.23 | 18.6±11.0 | 1.11 |
| 3 | CYP2C19 EM | clopidogrel | AM-H4 | 39.8±24.4 | 39.3±27.6 | 1.01 | 31.6±20.6 | 24.0±12.8 | 1.32 |
| 3 | CYP2C19 IM | clopidogrel | AM-H4 | 33.6±13.1 | 33.1±22.8 | 1.02 | 23.0±10.9 | 21.1±11.1 | 1.09 |
| 3 | CYP2C19 PM | clopidogrel | AM-H4 | 16.0±6.20 | 12.9±8.08 | 1.24 | 11.2±4.00 | 12.4±7.51 | 0.90 |
| 4 | NA | clopidogrel | AM-H4 | 9.83±4.42 | 10.2±8.49 | 0.96 | 9.71 | 7.13±5.56 | 1.36 |
| 4 | NA | vicagrel | AM-H4 | 6.23±2.34 | 6.62±2.76 | 0.94 | 8.95 | 6.72±3.22 | 1.33 |
| 4 | NA | vicagrel | AM-H4 | 12.2±4.35 | 13.2±5.50 | 0.92 | 12.48 | 13.2±5.50 | 0.95 |
| 4 | NA | vicagrel | AM-H4 | 22.5±10.1 | 19.7±8.22 | 1.17 | 23.62 | 19.9±9.57 | 1.19 |
| 4 | NA | vicagrel | AM-H4 | 47.9±17.8 | 39.0±16.3 | 1.22 | 55.68 | 39.2±19.0 | 1.42 |
| 5 | CYP2C19 EM | clopidogrel | AM-H4 | 29.6 | 27.0±16.7 | 1.10 | 22.5 | 16.9±5.51 | 1.34 |
| 5 | CYP2C19 IM | clopidogrel | AM-H4 | 19.2 | 22.9±13.9 | 0.84 | 15.1 | 14.2±4.95 | 1.06 |
| 5 | CYP2C19 PM | clopidogrel | AM-H4 | 12.6 | 10.2±7.03 | 1.24 | 8.53 | 9.39±2.21 | 0.91 |
| 5 | CYP2C19 EM | vicagrel | AM-H4 | 39.4 | 31.6±13.1 | 1.25 | 45.3 | 31.8±15.2 | 1.42 |
| 5 | CYP2C19 IM | vicagrel | AM-H4 | 31.3 | 31.3±13.0 | 1.00 | 37.4 | 31.4±15.1 | 1.19 |
| 5 | CYP2C19 PM | vicagrel | AM-H4 | 28.7 | 29.9±12.9 | 0.96 | 35.3 | 30.0±15.0 | 1.18 |
| *Parameters for the last dose* | | |  |  |  |  |  |  |  |
| 2 | NA | clopidogrel | clopidogrel | 2.88±4.23 | 2.44±1.81 | 1.18 | 1.77±3.37 | 1.33±1.63 | 1.33 |
| 2 | NA | clopidogrel | AM-H4 | 15.40±9.98 | 19.6±16.3 | 0.78 | 16.4±9.97 | 14.0±12.5 | 1.17 |
| 2 | NA | clopidogrel+omeprazole | clopidogrel | 4.00±4.58 | 2.84±2.21 | 1.41 | 1.98±2.95 | 1.48±1.90 | 1.34 |
| 2 | NA | clopidogrel+omeprazole | AM-H4 | 9.26±5.41 | 9.43±8.15 | 0.98 | 9.45±5.85 | 7.80±6.08 | 1.21 |
| 3 | CYP2C19 EM | clopidogrel | AM-H4 | 11.60±5.81 | 18.4±13.9 | 0.63 | 13.0±7.33 | 12.5±8.42 | 1.04 |
| 3 | CYP2C19 IM | clopidogrel | AM-H4 | 9.87±4.42 | 15.0±11.5 | 0.66 | 11.6±5.38 | 10.2±6.65 | 1.14 |
| 3 | CYP2C19 PM | clopidogrel | AM-H4 | 3.23±1.31 | 3.25±2.03 | 0.99 | 3.93±1.39 | 3.15±1.91 | 1.25 |
| 4 | NA | clopidogrel | AM-H4 | 6.50±2.98 | 10.2±8.49 | 0.64 | 6.03 | 7.13±5.56 | 0.85 |
| 4 | NA | vicagrel | AM-H4 | 5.84±2.00 | 6.62±2.76 | 0.88 | 9.68 | 6.72±3.22 | 1.44 |
| 4 | NA | vicagrel | AM-H4 | 11.10±4.46 | 13.2±5.50 | 0.84 | 9.07 | 13.2±5.50 | 0.69 |
| 4 | NA | vicagrel | AM-H4 | 16.70±6.03 | 19.7±8.22 | 0.85 | 16.25 | 19.9±9.57 | 0.82 |
| 4 | NA | vicagrel | AM-H4 | 10.30±4.45 | 9.91±4.13 | 1.04 | 10.99 | 10.1±4.82 | 1.09 |
| 5 | CYP2C19 EM | clopidogrel | AM-H4 | 9.38 | 12.4±8.27 | 0.75 | 9.58 | 8.45±5.32 | 1.13 |
| 5 | CYP2C19 IM | clopidogrel | AM-H4 | 5.41 | 8.10±6.68 | 0.54 | 5.59 | 6.94±4.29 | 0.81 |
| 5 | CYP2C19 PM | clopidogrel | AM-H4 | 4.03 | 2.58±1.79 | 1.56 | 3.95 | 2.46±1.91 | 1.60 |
| 5 | CYP2C19 EM | vicagrel | AM-H4 | 10 | 8.01±3.31 | 1.25 | 11.5 | 8.12±3.85 | 1.42 |
| 5 | CYP2C19 IM | vicagrel | AM-H4 | 8.08 | 7.91±3.30 | 1.02 | 12.5 | 8.03±3.84 | 1.56 |
| 5 | CYP2C19 PM | vicagrel | AM-H4 | 6.98 | 7.58±3.28 | 0.92 | 9.05 | 7.69±3.82 | 1.18 |

Parameters are presented as mean±SD when available.

EM: extensive metabolizers; IM: intermediate metabolizers; PM: poor metabolizers.

For trail 1, AUC_0-t_ refers to AUC_0-12_; For trail 2, AUC_0-t_ refers to AUC_0-24_; For trail 3, AUC_0-t_, t is unknown; For trail 4 and trail 5, AUC_0-t_ refers to AUC_0-4_.

Supplementary Table 4 Clinical study data used in verification of PBPK models of omeprazole and simvastatin and comparison to simulated results

| Drugs | Populations | Doses and regimens | Route | AUC_0-∞_ | | | C_max_ | | | References |
| --- | --- | --- | --- | --- | --- | --- | --- | --- | --- | --- |
|  |  |  |  | Observed | Simulated | Ratio of mean AUC_0-∞_ (Observed/ Simulated) | Observed | Simulated | Ratio of mean C_max_  (Observed/ Simulated) |  |
| omeprazole | Caucasian with *CYP2C19* **1/*1* genotype | 40 mg  single dose | oral | 4151 (2084, 6218) | 4043.63 (1130.49, 10883.27) | 1.03 | 2109 (1169, 3049) | 1635.56 (790.36, 2923.50) | 1.29 | (12) |
|  |  |  |  | Units were nM·h and nM, respectively. Parameters are presented as mean (95% CI). | | | | | | |
| simvastatin | Caucasian | 40 mg  single dose | oral | 25.2±16.6 | 28.0±22.5 | 0.90 | 6.87±3.30 | 7.83±5.42 | 0.88 | (13) |
|  |  |  |  | Units were ng/mL·h and ng/mL, respectively. Parameters are presented as mean±SD. | | | | | | |

Supplementary Table 5 Comparison of observed and simulated pharmacokinetic parameters of AM-H4 of Liu, C., et al.’s study (14)

| Subject number | Drugs | Doses and regimens | Route | AUC_0-t_ (ng·h/mL) | | Ratio of mean AUC_0-t_ (Observed/ Simulated) | C_max_ (ng/mL) | | Ratio of mean C_max_ (Observed/ Simulated) |
| --- | --- | --- | --- | --- | --- | --- | --- | --- | --- |
|  |  |  |  | Observed | Simulated |  | Observed | Simulated |  |
| 8 | clopidogrel | 75 single dose | oral | 11.8±4.94 | 10.2±8.49 | 1.16 | 9.93±4.74 | 7.13±5.56 | 1.39 |
| 12 | clopidogrel | 300 single dose | oral | 38.8±17.8 | 23.2±16.9 | 1.67 | 29.3±16.4 | 15.7±10.6 | 1.87 |
|  |  |  |  |  |  |  |  |  |  |
| 8 | vicagrel | 5 single dose | oral | 11.7±4.52 | 6.62±2.76 | 1.77 | 13.5±4.52 | 6.72±3.22 | 2.01 |
| 8 | vicagrel | 10 single dose | oral | 31.9±11.5 | 13.2±5.50 | 2.42 | 38.4±22.7 | 13.4±6.41 | 2.87 |
| 12 | vicagrel | 20 single dose | oral | 47.3±11.9 | 26.2±10.9 | 1.81 | 51.7±20.6 | 26.4±12.7 | 1.96 |
| 8 | vicagrel | 20 single dose | oral | 61.5±12.8 | 26.2±10.9 | 2.35 | 66.3±23.4 | 26.4±12.7 | 2.51 |
| 8 | vicagrel | 40 single dose | oral | 124±37.9 | 51.7±21.6 | 2.40 | 109±37.5 | 51.7±25.0 | 2.11 |
| 8 | vicagrel | 60 single dose | oral | 170±53.6 | 76.7±32.0 | 2.22 | 153±73.1 | 76.1±36.9 | 2.01 |
| 7 | vicagrel | 75 single dose | oral | 244±147 | 95.1±39.7 | 2.57 | 226±108 | 93.9±45.6 | 2.41 |

Parameters are presented as mean±SD.


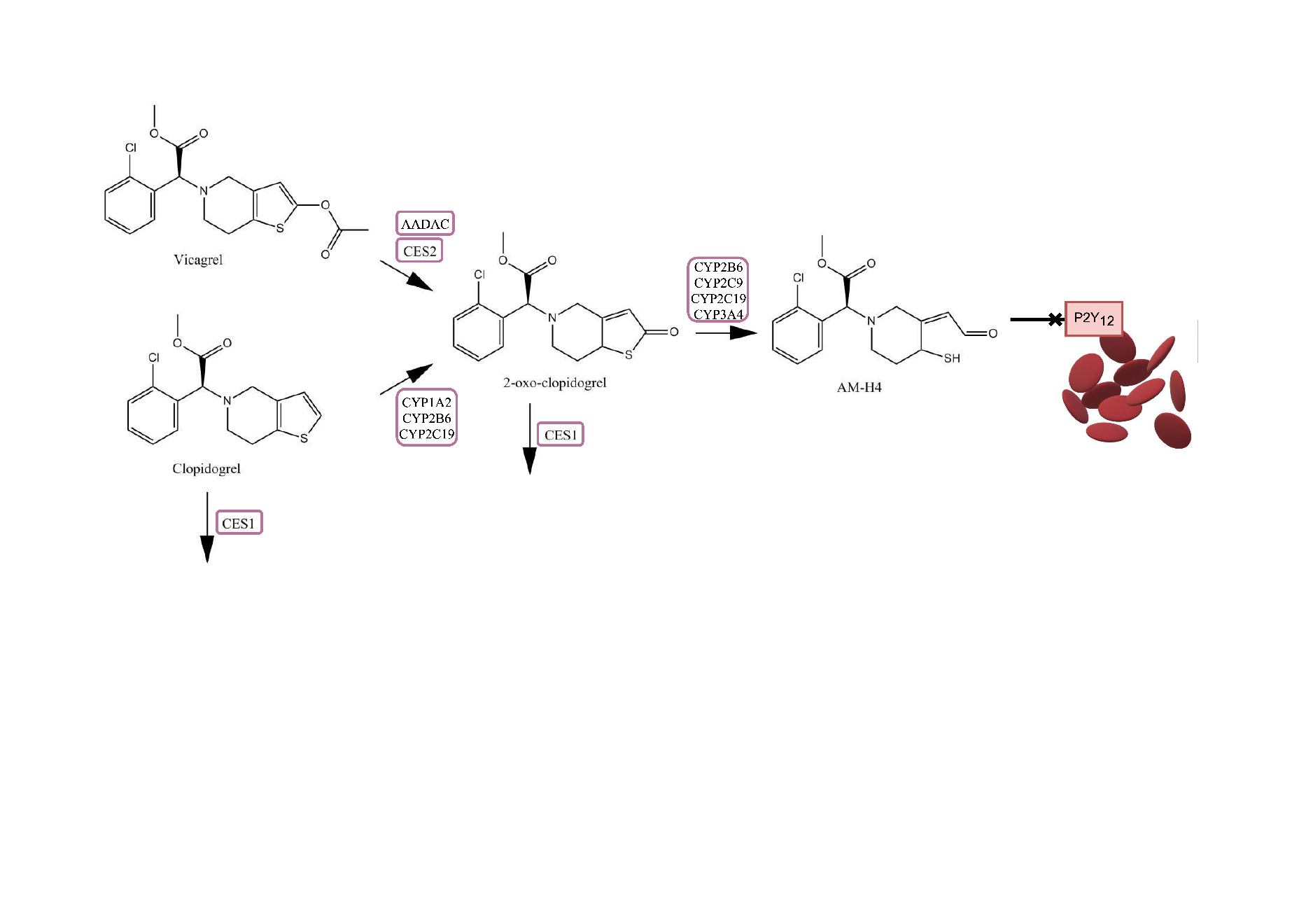


Supplementary Figure 1 Metabolic pathways of clopidogrel and vicagrel in humans.


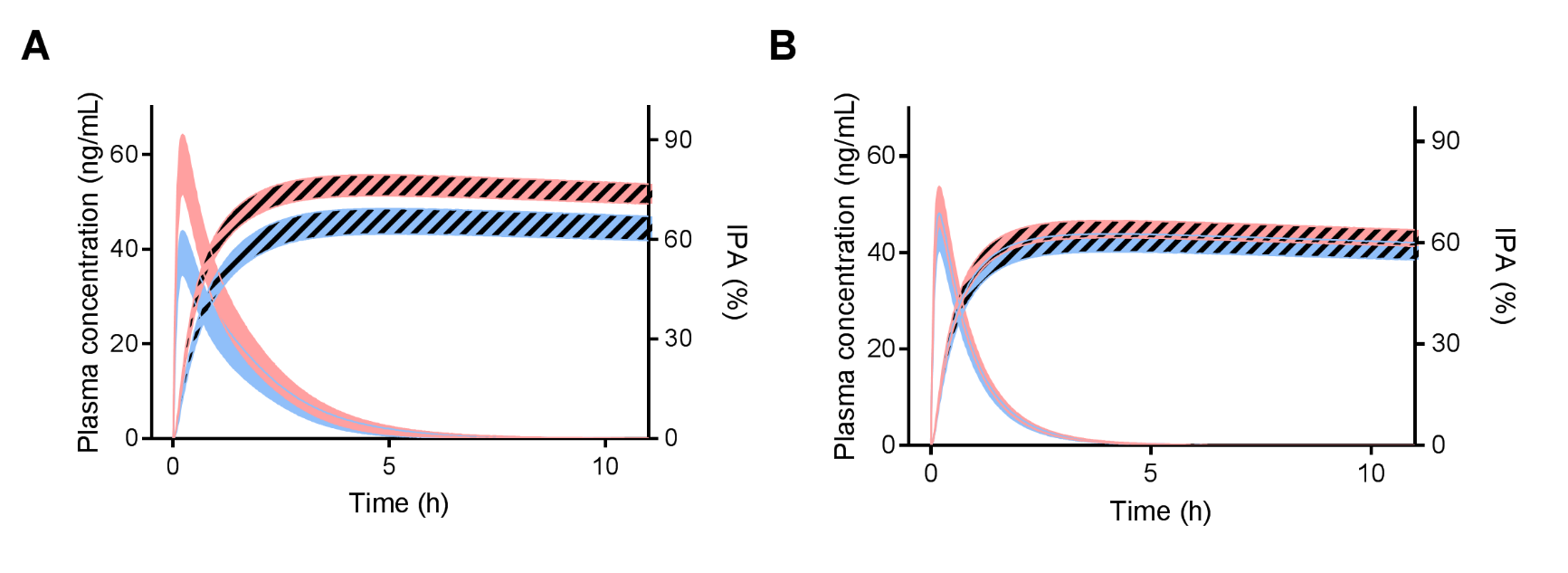


Supplementary Figure 2 Simulated AM-H4 concentration (left Y axis, single color) and IPA (right Y axis, color with shade) versus time of clopidogrel (A) and vicagrel (B) among volunteers carrying *CES1 428 G/G* (Red) or *G/A* (Blue) genotype. Dosage regimens were 300 mg LD and 75 mg/day MD for 4 days of clopidogrel (Trial 6) and 24 mg LD and 6 mg/day MD for 4 days of vicagrel, respectively.

**References**

1. Liu S, Wang Z, Ding X, Xu Q, Guo Z, Miao L. Determination of clopidogrel and its metabolites in plasma by UPLC-MS/MS and the application in pharmacokinetic study. *Chinese Journal of Pharmaceutical Analysis* (2015) 35(1):56-63.

2. Angiolillo DJ, Gibson CM, Cheng S, Ollier C, Nicolas O, Bergougnan L, et al. Differential effects of omeprazole and pantoprazole on the pharmacodynamics and pharmacokinetics of clopidogrel in healthy subjects: randomized, placebo-controlled, crossover comparison studies. *Clinical pharmacology and therapeutics* (2011) 89(1):65-74. Epub 2010/09/17. doi: 10.1038/clpt.2010.219. PubMed PMID: 20844485.

3. Simon T, Bhatt DL, Bergougnan L, Farenc C, Pearson K, Perrin L, et al. Genetic polymorphisms and the impact of a higher clopidogrel dose regimen on active metabolite exposure and antiplatelet response in healthy subjects. *Clinical pharmacology and therapeutics* (2011) 90(2):287-95. Epub 2011/07/01. doi: 10.1038/clpt.2011.127. PubMed PMID: 21716274.

4. Li X, Liu C, Zhu X, Wei H, Zhang H, Chen H, et al. Evaluation of Tolerability, Pharmacokinetics and Pharmacodynamics of Vicagrel, a Novel P2Y12 Antagonist, in Healthy Chinese Volunteers. *Front Pharmacol* (2018) 9:643. Epub 2018/07/06. doi: 10.3389/fphar.2018.00643. PubMed PMID: 29973877; PubMed Central PMCID: PMCPMC6019484.

5. Zhang Y, Zhu X, Zhan Y, Li X, Liu C, Zhu Y, et al. Impacts of CYP2C19 genetic polymorphisms on bioavailability and effect on platelet adhesion of vicagrel, a novel thienopyridine P2Y12 inhibitor. *British journal of clinical pharmacology* (2020). Epub 2020/04/09. doi: 10.1111/bcp.14296. PubMed PMID: 32267573.

6. Tarkiainen EK, Holmberg MT, Tornio A, Neuvonen M, Neuvonen PJ, Backman JT, et al. Carboxylesterase 1 c.428G>A single nucleotide variation increases the antiplatelet effects of clopidogrel by reducing its hydrolysis in humans. *Clinical pharmacology and therapeutics* (2015) 97(6):650-8. Epub 2015/02/24. doi: 10.1002/cpt.101. PubMed PMID: 25704243.

7. Tornio A, Filppula AM, Kailari O, Neuvonen M, Nyrönen TH, Tapaninen T, et al. Glucuronidation converts clopidogrel to a strong time-dependent inhibitor of CYP2C8: a phase II metabolite as a perpetrator of drug-drug interactions. *Clinical pharmacology and therapeutics* (2014) 96(4):498-507. Epub 2014/06/28. doi: 10.1038/clpt.2014.141. PubMed PMID: 24971633.

8. Zhu HJ, Wang X, Gawronski BE, Brinda BJ, Angiolillo DJ, Markowitz JS. Carboxylesterase 1 as a determinant of clopidogrel metabolism and activation. *The Journal of pharmacology and experimental therapeutics* (2013) 344(3):665-72. Epub 2013/01/01. doi: 10.1124/jpet.112.201640. PubMed PMID: 23275066.

9. Jiang J, Chen X, Zhong D. Arylacetamide Deacetylase Is Involved in Vicagrel Bioactivation in Humans. *Front Pharmacol* (2017) 8:846. Epub 2017/12/07. doi: 10.3389/fphar.2017.00846. PubMed PMID: 29209217; PubMed Central PMCID: PMCPMC5701912.

10. Jiang XL, Samant S, Lewis JP, Horenstein RB, Shuldiner AR, Yerges-Armstrong LM, et al. Development of a physiology-directed population pharmacokinetic and pharmacodynamic model for characterizing the impact of genetic and demographic factors on clopidogrel response in healthy adults. *Eur J Pharm Sci* (2016) 82:64-78. Epub 2015/11/03. doi: 10.1016/j.ejps.2015.10.024. PubMed PMID: 26524713; PubMed Central PMCID: PMCPMC5798599.

11. Djebli N, Fabre D, Boulenc X, Fabre G, Sultan E, Hurbin F. Physiologically based pharmacokinetic modeling for sequential metabolism: effect of CYP2C19 genetic polymorphism on clopidogrel and clopidogrel active metabolite pharmacokinetics. *Drug metabolism and disposition: the biological fate of chemicals* (2015) 43(4):510-22. Epub 2015/01/23. doi: 10.1124/dmd.114.062596. PubMed PMID: 25609219.

12. Baldwin RM, Ohlsson S, Pedersen RS, Mwinyi J, Ingelman-Sundberg M, Eliasson E, et al. Increased omeprazole metabolism in carriers of the CYP2C19*17 allele; a pharmacokinetic study in healthy volunteers. *British journal of clinical pharmacology* (2008) 65(5):767-74. Epub 2008/02/26. doi: 10.1111/j.1365-2125.2008.03104.x. PubMed PMID: 18294333; PubMed Central PMCID: PMCPMC2432489.

13. Backman JT, Kyrklund C, Kivistö KT, Wang JS, Neuvonen PJ. Plasma concentrations of active simvastatin acid are increased by gemfibrozil. *Clinical pharmacology and therapeutics* (2000) 68(2):122-9. Epub 2000/09/08. doi: 10.1067/mcp.2000.108507. PubMed PMID: 10976543.

14. Liu C, Zhang Y, Chen W, Lu Y, Li W, Liu Y, et al. Pharmacokinetics and pharmacokinetic/pharmacodynamic relationship of vicagrel, a novel thienopyridine P2Y12 inhibitor, compared with clopidogrel in healthy Chinese subjects following single oral dosing. *Eur J Pharm Sci* (2019) 127:151-60. Epub 2018/10/17. doi: 10.1016/j.ejps.2018.10.011. PubMed PMID: 30326264.
